# Comparative Study of the Quality of Life of Patients With Ototoxicity Due to Platinum-Based Chemotherapy, With Hearing Aid Versus Those Without: Study Protocol for a Randomized Pilot Study—The PROTOTOX Study

**DOI:** 10.2196/71562

**Published:** 2025-10-10

**Authors:** Romina Mastronicola, Elise Kayser, Yolanda Fernandez, Gilles Dolivet

**Affiliations:** 1 Institut de Cancérologie de Lorraine, 6 avenue de Bourgogne, Vandœuvre-lès-Nancy, 54500, France, 33 383598400; 2 Centre de Recherche en Automatique de Nancy (CRAN), UMR 7039, CNRS, Université de Lorraine, Vandœuvre-lès-Nancy, France

**Keywords:** chemotherapy, equipment, hearing aid, hypoacusis, platinum salts

## Abstract

**Background:**

Platinum salts are widely used for the treatment of cancers, including head and neck cancers. Despite their efficacy, platinum salts can induce neurosensory disorders such as ototoxicity, tinnitus, and decreased hearing acuity. These side effects can have a major impact on the quality of life of patients and are not often considered after treatment.

**Objective:**

The PROTOTOX study aims to compare the quality of life of patients with hearing aids in the case of ototoxicity due to platinum salt-based chemotherapy, according to 2 treatments: standard management without equipment versus standard management with equipment.

**Methods:**

The PROTOTOX study is an open pilot prospective monocentric and randomized study executed through collaboration of the Institut de Cancérologie de Lorraine (nonprofit comprehensive cancer institute). A total of 52 patients with head and neck cancer undergoing platinum-based chemotherapy and presenting with hypoacusis will be included and followed by an ear, nose, and throat specialist. Audiometric testing will be performed, and eligible participants who consent will be randomized to either receive or not receive hearing aids (groups 1 and 2). The primary endpoint of this study is to compare the quality of life of the patients undergoing platinum-based chemotherapy presenting with hypoacusis according to 2 ways of care: standard care without hearing aid versus standard care with hearing aid. Secondary outcomes are the evaluation of the hearing and the tinnitus, the evaluation of the patients’ satisfaction at the end of the study, and the evaluation of the patients’ adherence to hearing aid in case of ototoxicity due to platinum-based chemotherapy.

**Results:**

The study protocol has been opened and is actively recruiting participants. The protocol was launched on November 23, 2023, and data collection is ongoing and will be completed once all patients have completed the protocol. At least 27 patients have been recruited to date. The date of publication of the results is not yet known.

**Conclusions:**

The PROTOTOX study aims at demonstrating that the huge impact of platinum-based chemotherapy on hearing abilities must be managed to maintain the patients’ quality of life. Hearing aid is the solution experimented here.

## Introduction

### Background

Platinum salts (cisplatin, carboplatin, and oxaliplatin) are alkylating agents widely used for the treatment of several cancers: bladder, head and neck, lungs, ovary, cervix, endometrium, breast, brain, thyroid, bone, and testes. Although very effective, the adverse effects of platinum-based cancer treatments are numerous: kidney failure; thrombocytopenia; leukopenia; peripheral neuropathies; vomiting; nausea; and decreased plasma levels of magnesium, calcium, and potassium. In addition, the administration of anticancer treatments based on platinum salts induces neurosensory disorders such as ototoxicity, tinnitus, and decreased hearing acuity.

Hearing loss is very common (≥1/10) and is found in approximately 31% of patients treated with a dose of 50 mg cisplatin m^–^² [1]. The ototoxic potential of cisplatin puts patients at risk of hearing loss and leads to additional negative impact on the patients’ quality of life. Administration of cisplatin causes ototoxicity with a highly variable incidence rate. This variability depends on the duration of treatment, the dose received, the method of administration, and the treatment of the population. Textbox 1 shows some examples of the incidence rates observed depending on the treatment and dose and the audiology test performed [2].

Textbox 1. **Incidence rates observed depended on the treatment, dose, and the audiology test performed.**

- 100% of 34 patients with head and neck cancers receiving cisplatin-containing chemotherapy and concomitant radiotherapy
- 55.1% of 107 patients receiving cisplatin-containing chemotherapy, regardless of cancer type
- 31% up to 8 kHz and 47% up to 16 kHz with air and bone conduction tone audiometry (0.125-16 kHz) of 60 patients with locally advanced head and neck cancer, receiving cisplatin-containing chemotherapy and concomitant radiotherapy
- 15% of 60 patients receiving cisplatin-containing chemotherapy: 12% of 51 patients in the low-dose group; 33% of 9 patients in the high-dose group

A significant component of the acute hearing loss seen in patients undergoing chemotherapy relates to local inflammation, middle ear effusions, and eustachian tube obstruction. This tends to improve significantly, even resolving fully, over time as the inflammation resolves.

The cytotoxic effect of cisplatin is dose-dependent, and therefore less significant in multidrug therapy treatments, depending on the dosing used in the regimen [3]. There are multiagents that use cisplatin at a high dose. It is higher in patients receiving cumulative doses [4] or in a pediatric population [5]. The ototoxicity of cisplatin is often bilateral, symmetrical, and irreversible, mainly affecting high frequencies (4000-8000 Hz), whether or not associated with tinnitus [2]. Hearing impairment at frequencies between 250 and 6000 Hz (normal hearing range) is measured in 10%-15% of patients treated with cisplatin [6]. In rare cases, unilateral hearing loss has been reported depending on the location of the tumor and associated treatments [7]. Hearing loss caused by platinum agents is permanent [8].

Single or bilateral, ototoxicity becomes more frequent and more severe with the intensity and repetition of doses. It is increased by prior hearing loss and association with ototoxic drugs (loop diuretics like furosemide, or aminoglycoside antibiotics like gentamicin). Patients receiving carboplatin and oxaliplatin have lower ototoxicity, ranging from 15% of patients, with abnormalities located at the high frequencies (4000‐8000 Hz).

A recent study estimated the prevalence of ototoxicity and its associated risk factors in a cohort of 145 testicular cancer survivors who received cisplatin-based first-line chemotherapy [9]. About 74% reported ototoxicity: 68% tinnitus, 59% hearing loss, and 52% both. Survivors with tinnitus were more likely to report hypercholesterolemia (*P*=.008) and hearing difficulties (*P*<.001). Tinnitus was also significantly related to age at the time of the survey (OR=1.79; *P*=.003) and the cumulative dose of cisplatin (OR=5.17; *P*<.001). Risk factors for hearing loss included age at the time of the survey (OR=1.57; *P*=.04), hypercholesterolemia (OR=3.45; *P*=.007), cumulative cisplatin dose (OR=1.94; *P*=.049) and family history of hearing loss (OR=2.87; *P*=.07). In conclusion, the authors recommend referring patients to audiologists before, during, and after cisplatin treatment.

Since then, clinical trials have been developed by offering drugs related to supportive care to limit the cytotoxic effect of cisplatin. Another proposes a thiosulfate treatment that results in a reduction in the neoplastic activity of cisplatin but does not show a significant result [10]. A study conducted in rats showed a decrease in the ototoxicity of cisplatin with the administration of a preventive treatment of diethyldithiocarbamate [11].

One review addressed 11 studies that looked at quality of life and chemotherapy-induced toxicities, such as hearing loss and tinnitus [12]. Overall, the results revealed that people treated with platinum-based chemotherapy, particularly cisplatin, had significantly more hearing loss and tinnitus than the population who did not receive it, and higher doses were correlated with persistent symptoms. These results are consistent with those in the literature as it is reported that, on average, 60%‐70% of adult patients experienced ototoxicity when treated with cisplatin [13-16]. This review found that people with tinnitus and hearing loss were more likely to have a lower quality of life.

According to an INSERM survey carried out in 2018 [17], hearing impairment due to cancer treatments is estimated at 39.7% of respondents, 5 years after a cancer diagnosis. A study summarized the impact of the long-term effects of hearing loss, tinnitus, and balance in children and adults treated with platinum-based chemotherapy [18]. The authors conclude that ototoxicity can lead to communication difficulties, subsequent social withdrawal, impaired cognitive functions, and repercussions on professional activity. Ototoxicity was linked to deficits in cognition, child development, and school performance. Employment and ease of daily life were disrupted by deafness and tinnitus. Depression and anxiety were linked to ototoxicity. The authors advocate early intervention for better management of ototoxicity, thereby improving the quality of life of affected patients. Similarly, tinnitus is associated with increased anxiety, depression, insomnia, and decreased concentration [19-22].

There is a clinical study on the ototoxic effects of chemotherapy on patients’ quality of life [23]. In this study, 20 patients were asked about their perception of the ototoxic effect with semistructured interviews based on 2 main themes (ototoxicity-related quality of life and cancer-related quality of life). Subthemes included the impact of ototoxicity, hearing, tinnitus, clinical experience, audiological assessments, and the impact of treatment, cancer and chemotherapy, other toxicities, patient information, and reflections. Recruited patients describe fear of worsening symptoms, tinnitus-related fatigue, reduced sleep, and frustration. We can also note a decrease in their social interactions. It is described that patients would have liked to have had more information and follow-up on the ototoxicity of their chemotherapy. The study concludes that ototoxicity can have a negative impact on quality of life, especially on social life and fear of hearing loss and worsening tinnitus. The authors also report a lack of awareness among patients and clinicians about auditory monitoring and the management of patients receiving platinum-based chemotherapy. The main limitation of this study is that medical history was declared by the patients themselves and not by a collection of medical records due to lack of access. This can lead to unreliable data. The authors raised the issue where participants could not remember the type of chemotherapy they had received. Thus, their hearing loss could be related to age or noise, and not to ototoxic chemotherapy.

### Need for the Study

An observational case-control study analyzed the audiological outcomes and quality of life of cancer and noncancer patients [24]. In the oncology group, 32 participants had to have an anatomopathological diagnosis of neoplasia and who had undergone radiotherapy and chemotherapy with cisplatin. Hearing loss was assessed by conventional tonal audiological examination and quality of life by the Medical Outcomes Study 36-Item Short-Form Health Survey (SF-36). It is a multidimensional questionnaire consisting of 36 questions divided into 8 domains: functional capacity, physical aspects, pain, general health, vitality, social aspects, emotional aspects, and mental health. The results were assessed by assigning scores for each question, which were transformed on a scale of 0-100, where 0 corresponded to a poorer quality of life and 100 to a better quality of life. The results indicate that 35.2% of patients had hearing loss in the oncology group. Textbox 2 shows the results of the analysis of the SF-36 questionnaires yielded scores.

Textbox 2. **Results of the analysis of the 36-Item Short-Form Health Survey (SF-36) questionnaires yielded scores.**

- 70.47: functional ability
- 56.25: physical aspects
- 48.91: pain
- 53.28: general health
- 40.69: vitality
- 72.13: social aspects
- 48.91: emotional aspects
- 49.44: mental health

A change greater than 5 points on a SF-36 subscale is generally considered clinically meaningful, indicating a noticeable improvement or decline in a patient’s health status. Clinically, such a change may reflect enhanced physical functioning (eg, easier walking or daily activities), reduced pain, increased vitality, improved emotional well-being, or greater social participation. It represents a level of change that patients perceive as beneficial or impactful in their daily lives, beyond mere statistical significance. In a perfectly functional state, individuals typically score between 85 and 100 across all domains, reflecting full physical, emotional, and social well-being. A limited state, such as in chronic but manageable conditions, yields scores between 40 and 70, indicating moderate impairments in areas such as mobility, pain, or vitality. In contrast, a disabled or severely impaired state is characterized by scores below 40 in multiple domains, reflecting significant physical limitations, chronic pain, poor mental health, and reduced quality of life.

The SF-36 quality of life score is significantly different from the control group in these two domains: general health and mental health.

Unlike previous studies that focused primarily on pharmacological otoprotectants, which often present limitations in efficacy or interfere with chemotherapy’s antitumor effects, this study explores a noninvasive, supportive approach through the early, systematic use of hearing aids—an area that remains largely unstudied. To date, no clinical trial has specifically evaluated the impact of proactive hearing aid use on quality of life outcomes in patients receiving platinum-based chemotherapy, despite growing evidence of the burden of ototoxicity. This pilot study addresses a critical gap by investigating a practical, immediately deployable intervention to manage chemotherapy-induced hearing loss, aiming to improve adherence and patient well-being without altering oncologic treatment protocols [25,26].

Our hypothesis is that a systematic hearing aid for patients suffering from ototoxicity of chemotherapy from the start of treatment would bring a benefit to these patients, by improving their quality of life. A hearing aid is a noninvasive intervention, and that is why we choose it over other interventions. The improvement in patients’ quality of life is assessed by the SF-36 quality of life questionnaire. This questionnaire was used in a study to demonstrate a significant relationship between emotional aspects and the presence of tinnitus as well as hearing loss [24].

Regarding the collective benefit of this study, the generalization of the patients’ equipment during their chemotherapy protocol would allow an increase in quality of life and better adherence to treatment.

### Objectives

The primary objective of the PROTOTOX study is to compare the quality of life of patients presenting with ototoxicity due to platinum-based chemotherapy following 2 different ways of care: standard management without hearing aid versus standard management with hearing aid. The secondary objectives are (1) to evaluate and compare hearing and tinnitus in patients with hearing aid and ototoxicity induced by platinum-based chemotherapy and patient satisfaction at the end of the study in the 2 groups (control and experimental), (2) to assess patients’ adherence to hearing aid in case of ototoxicity due to platinum-based chemotherapy.

## Methods

The PROTOTOX study is an open and prospective interventional monocentric pilot study with minimal risks and constraints. The study is registered on French Competent Authority (ID-RCB: 2023-A02141-44) and ClinicalTrials.gov (NCT05936034). The study is randomized into the control group, that is standard care without hearing aids, and the experimental group, standard care with hearing aids.

Standard care will entail an audiometric test and classical control visits.

### Ethical Considerations

#### Regulatory Requirements

The study will be conducted in accordance with the ethical principles of the latest version of the Declaration of Helsinki, The Good Clinical Practice of 24 November 2006 defined by the International Conference on Harmonization (ICH-E6) [27], the Data Protection Act No. 78‐17 of 6 January 1978 as amended by Law No. 2004-801 of 6 August 6, 2004 on the protection of individuals regarding the processing of personal data [28], The Bioethics Law No. 2004‐800 of 6 August 2004 [29], Decree No. 2016‐1537 of 16 November 2016 on research involving human beings [30], the Decree of 7 May 2017 establishing the list of research mentioned in 2° of Article L. 1121‐1 of the Public Health Code [31], and the Decree of 9 May 2017 amending certain regulatory provisions relating to research involving human beings [32].

- Data Protection Act No. 78‐17 of 6 January 1978 as amended by Law No. 2004-801 of 6 August 6, 2004 on the protection of individuals regarding the processing of personal data [28]
- The Bioethics Law No. 2004‐800 of 6 August 2004 [29]
- Decree No. 2016‐1537 of 16 November 2016 on research involving human beings [30]
- Decree of 7 May 2017 establishing the list of research mentioned in 2° of Article L. 1121‐1 of the Public Health Code [31]
- Decree of 9 May 2017 amending certain regulatory provisions relating to research involving human beings [32]

#### Ethics Committee, Competent Authority, and CNIL (National Data Privacy Commission)

Before carrying out research on human beings, the sponsor is required to submit the project for approval to an Ethics Committee with the competence for the location in which the lead investigator practices and for information to the French health product safety agency (ANSM). The request for an opinion on the intervention research project with minimal risks and constraints is sent to the committee and the competent authority by the sponsor.

As such, the protocol was submitted to the Ethics Committee (Comité de Protection des Personnes Sud Est I) on November 11, 2023, which issued a favorable opinion (2023-A02141-44) and was sent to the ANSM for information, No. 23.03486.000352.

Requests for substantial changes to the initial projects will also be sent by the promoter to the committee for its opinion and to the competent authority for information.

The study complies with the MR-001 methodology published by CNIL (national data privacy commission) [33].

#### Insurance

In accordance with the current legislation of the Public Health Code [34], the sponsor (Institut de Cancérologie de Lorraine) has maintained liability insurance for the duration of the study to cover its liabilities and those of any other participating parties in accordance with the regulations in force under number B1339CTLICNWL22-73 with Lloyd’s Insurance Company.

#### Patient Information and Informed Consent

Prior to carrying out intervention research with minimal risks and constraints on a person, the free, informed, and express consent (written or oral) of the person must be obtained after he or she has been exhaustively informed, by the investigator, during a consultation and after a sufficient period of reflection. The sponsor may ask the person participating in research at the time the person gives his or her informed consent when it is required to agree to the use of his or her data in subsequent research exclusively for scientific purposes. The person may withdraw his or her consent to this further use or exercise his or her right to object at any time.

Similarly, if an amendment to the protocol were to lead to a revision of the information forms intended for patients participating in the research and if it requires the collection of new consent, the request sent to the Ethics Committee concerned for opinion shall include a description of the procedures envisaged for obtaining this new consent.

For tests intended to perform genomics or proteomics analyses, the information form must specify the type of research that will be conducted. The use of these samples for a medical or scientific purpose other than that for which they were taken or collected is possible, unless the person from whom the sampling or collection was carried out, duly informed in advance of this other purpose, objects. The obligation to provide information may be waived when it is impossible to find the person concerned, or when one of the advisory Ethics Committees (Comité de Protection des Personnes) referred to in Article L. 1123‐1, consulted by the person responsible for the investigation, does not consider this information necessary [35].

This personal information must be kept in the file of the participating physician and treated in strict confidentiality but must be reviewable by the appropriate authorities and duly authorized people.

According to Article 1122-1-1, if the person taking part in research has withdrawn his or her consent, this withdrawal does not affect the activities carried out and the use of the data obtained based on the informed consent expressed before it was withdrawn [36].

### Study Population

The PROTOTOX study is proposed to patients with head and neck cancer managed by a platinum-based chemotherapy at the Institut de Cancérologie de Lorraine. Textbox 3 includes the inclusion and exclusion criteria.

Textbox 3. **Inclusion and exclusion criteria.**

**Inclusion criteria**

- Patient aged 18 years or older
- Patient on treatment with platinum-based chemotherapy, with treatment-consistent hearing loss or with a worsening of existing hearing loss consistent with the start of platinum-based chemotherapy treatment
- Patient whose hearing loss is confirmed by audiometric testing
- Patient is able to and willing to follow all study procedures in accordance with the protocol
- Patient who understood, signed, and dated the consent form
- Patient affiliated with the social security scheme

**Noninclusion criteria**

- Pregnant or breastfeeding woman
- Persons deprived of liberty or under guardianship (including curatorship)
- Inability to undergo medical monitoring of the trial for geographical, social, or psychological reasons
- Patient with a contraindication to wearing hearing aid
- Patient already fitted with a hearing aid
- Patient already included in a protocol including an experimental molecule
- Patient who has not started treatment with platinum-based chemotherapy
- Tinnitus-only patient without hearing loss

### Sample Size Calculation

A study estimated a mean quality of life score by the SF-36 questionnaire at 55 with a standard deviation of 20 in a population of 32 patients where the ototoxicity rate was 35.2% [24]. In our population with 100% ototoxicity, the expected SF-36 score in the control group would be lower (mean score less than 55) and less variable (standard deviation less than 20).

The justification for the number of subjects required is based on the size of the anticipated effect [37]. A comparison of the two groups (control and experimental) via an analysis of covariance (ANCOVA) adjusted to the baseline (estimated scores at baseline) will be performed to meet the primary objective.

Under the assumption of a common standard deviation of 15 points, an intra-group correlation coefficient *r*=0.8 (correlation between baseline and follow-up measures) [38], a statistical power level of 80%, and an alpha risk of 5%, a sample size of 26 patients per group will detect a minimum difference of 7 points between the control and experimental groups (7/15=0.46, an average effect size). If the effect size is smaller (difference less than 7 points), the results of the ANCOVA model will therefore not be statistically significant. In this case, we can use SF-36 estimates to accurately calculate the number of subjects for a larger study.

### Statistical Analysis

Clinical-pathological characteristics of the patient, SF-36 scores, audiometric/acouphenometric measures, and patient satisfaction will be described in each group (control and experimental), in Baseline, M1, M3, and M6. Quantitative parameters will be described by mean (SD), median, min–max, and 1st and 3rd quartiles. Qualitative parameters will be described by frequency and percentage.

To meet the main objective, the overall SF-36 score of quality of life will be described in each group, and the normality of the data will be tested by the Shapiro-Wilk method. A comparison of the scores of the 2 groups in baseline and follow-up at the first month (M1), third month (M3), and sixth month (M6) via a Student *t* test will be used if the normality of the distribution has been verified; otherwise, a Wilcoxon Mann-Whitney *U* test will be used.

An ANCOVA will be performed to compare the scores of the 2 groups at the M1, M3, and M6 follow-ups by adjusting on the observed scores in baseline (initial state of the patient score). Given the limited sample size, stratification will be performed only on the type of treatment, which is most likely to influence both the severity of ototoxicity and the response to hearing rehabilitation. Other variables such as cancer type and cumulative dose will be included as covariates in the ANCOVA model or explored in sensitivity analyses.

To meet secondary objectives, the patients’ hearing will be characterized by a binary qualitative variable (worsening or stagnation). It will be described in each group according to follow-up in M1, M3, and M6 and will be compared by a *χ*^2^ test. The same analysis will be performed for the appearance of tinnitus measured by an incidence rate. A Student test will be used to compare patient satisfaction from the 2 groups if the data are normally distributed; otherwise, a Wilcoxon Mann-Whitney test will be used. The adherence rate is described by the proportion of patients who completed the 6 months of protocol follow-up with the wearing of the hearing aid among the patients randomized to the experimental group. Censored data (patients who died or were lost to follow-up before the end of follow-up) will be considered in estimating patient adherence rates.

Statistical analyses will be performed using RStudio software version 2022.07.2+576 (developed by Joseph Allaire). The materiality threshold is set at 5%.

### Study Procedures

#### Information and Consent

Patients coming to the Institut de Cancérologie de Lorraine for cancer treatment with platinum-based chemotherapy and presenting with hearing loss will be referred to a clinical ear, nose, and throat (ENT) specialist (principal investigator of the study).

The ENT specialist will suggest that the patient undergo an audiometric and tinnitus test to confirm the hearing loss. If the audiometric and tinnitus test confirms the patient’s hearing loss, and if all other eligibility criteria are verified, the principal investigator will present the PROTOTOX study to the patient.

After a period of reflection, the patient’s free, informed, and signed consent will be collected, and the patient will be included in the study. Patients whose audiometric and tinnitus tests do not confirm hypoacusis will continue their usual care.

#### Inclusion and Randomization

Patients will be included in the study via the CleanWeb website. After signing consent and completing the V0 baseline visit, they will be randomly assigned (1:1 simple randomization) to the control or experimental group.

The inclusion assessment (V0) includes (Textbox 4):

- Control group: standard care without hearing aid
- Experimental group: standard care with hearing aid

Textbox 4. **Proceedings of the inclusion assessment.**

For all patients:

- A consultation with an ear, nose, and throat (ENT) specialist at the institute with an audiometric assessment carried out by an audiogram and a tinnitus assessment, according to the recommendations related to the treatment
- The collection of the 36-Item Short-Form Health Survey (SF-36) quality of life questionnaire completed by the patient
- The collection of treatments received with platinum salt: molecules, frequency, doses, and an inquiry about other ototoxic medications
- The collection of adverse events greater than 2 or having a significant impact on treatment (these include renal function and electrolyte derangements)
- The date of signing of consent and date of inclusion
- Randomization date and randomization arm

For experimental group only:

- The reference number of the hearing aid provided to the patient

Patients in the experimental group will be referred to certified hearing aid specialists from a preapproved network, who will follow a standardized fitting protocol, including device calibration, patient instruction, and adjustment follow-ups. Systematic use will be encouraged, targeting at least 6 hours/day, and adherence will be self-reported and recorded at each follow-up.

Simple randomization was chosen due to the exploratory nature and small sample size (n=52) of this pilot study, which primarily aims to assess feasibility and preliminary effects. While we acknowledge the risk of imbalance in key prognostic factors (eg, age, baseline hearing, cumulative cisplatin dose, prior ototoxic drugs), these variables will be collected and adjusted for in the planned ANCOVA analyses. Stratified or minimization techniques will be considered in future larger trials to enhance group comparability.

#### Follow-Up Visits

All follow-up visits will be scheduled during those taking place as part of the care in order to avoid the patient having to travel specifically for research.

Follow-up visits (V1 and V2) are carried out by the institute’s clinical ENT specialist: for patients in the control group at 1 (V1) and 3 (V2) months after randomization and for patients in the experimental group at 1 (V1) and 3 (V2) months after randomization and equipment (M1 and M3).

During these visits, and for all patients in the study, the institute’s clinical ENT specialist will perform a clinical examination and an audiometric and tinnitus examination. The patient will complete the SF-36 quality of life questionnaire.

The follow-up reports (V1 and V2) are included in Textbox 5.

Textbox 5. **Proceedings of the follow-up reports.**

For all patients:

- An ear, nose, and throat (ENT) consultation with an ENT specialist from the institute with an audiometric assessment carried out by an audiogram and a tinnitus assessment, according to the recommendations related to the treatment
- The collection of the 36-Item Short-Form Health Survey (SF-36) quality of life questionnaire completed by the patient
- The collection of treatments received with platinum salt: molecule, frequency, and doses
- The collection of adverse events greater than 2 or having a significant impact on treatment

For the experimental group only:

- The collection of the time the hearing aid has been worn
- Adverse effects related to the wearing of the hearing aid: damage to the ear canal, embarrassment, autophony, occlusion, earwax plug, and need for hearing aid adjustment
- Possible dysfunctions of the hearing aid

#### End-of-Study Visit

The end-of-study visit (V3) is carried out by the institute’s clinical ENT specialist: for patients in the control group 6 months after randomization and for patients in the experimental group 6 months after equipment (M6).

This visit will be scheduled when the patient arrives as part of his/her standard care.

During this visit, and for all patients in the study, the institute’s clinical ENT specialist performs a clinical examination and an audiometric and tinnitus examination of the patient; the patient will complete the SF-36 quality of life questionnaire and a satisfaction questionnaire. At the end of the V3 visit, patients in group 2 only will give the hearing aid back to the clinician.

Following this visit, all patients are discharged from the study and go on with their care according to the recommendations specific to their pathology.

The end-of-treatment assessment (V3) is included in Textbox 6.

Textbox 6. **Proceedings of the end-of-treatment assessment.**

For all patients:

- An ear, nose, and throat (ENT) consultation with an ENT specialist from the institute with an audiometric assessment carried out by an audiogram and a tinnitus assessment, according to the recommendations related to the treatment
- The collection of the 36-Item Short-Form Health Survey (SF-36) quality of life questionnaire completed by the patient
- The collection of treatments received with platinum salt: molecule, frequency, and doses
- The collection of adverse events greater than 2 or having a significant impact on treatment

For the experimental group only:

- The collection of the time the hearing aid has been worn
- Adverse effects related to wearing the hearing aid: damage to the ear canal, embarrassment, autophony, occlusion, earwax plug, and need for hearing aid adjustment
- Possible dysfunctions of the hearing aid

#### Description of the SF-36 and Satisfaction Questionnaires

The quality of life questionnaire is based on the SF-36 self-questionnaire [39]. The items in SF-36 are divided into 8 different areas (Textbox 7).

Except for questions about the general change in health status, patients are asked to answer based on the last 4 weeks.

The SF-36 is a validated and widely used instrument for assessing health-related quality of life across diverse patient populations. Its inclusion in this clinical research protocol is justified by its ability to capture a broad range of physical, emotional, and social health domains through 8 subscales, including physical functioning, role limitations, bodily pain, vitality, general health perceptions, and mental well-being. This multidimensional structure allows for a comprehensive evaluation of the patient’s perceived health status, which is especially relevant in conditions such as ototoxicity, where functional impairments may not be life-threatening but can significantly impact daily life. Moreover, the SF-36 is sensitive to clinical changes over time and enables the detection of both improvements and deteriorations in quality of life following an intervention, such as the use of hearing aids. Its standardized format also facilitates comparisons with normative data or across studies. Finally, the ability to compute both physical and mental component summary scores provides flexibility for both global and domain-specific analysis, making the SF-36 a robust and appropriate tool for evaluating the impact of this intervention on patients’ overall well-being [40].

The satisfaction questionnaire is based on a numerical scale starting at 0 (not satisfied) to 10 (very satisfied).

Textbox 7. **Items of the 36-Item Short-Form Health Survey (SF-36) questionnaire.**

Physical component:

- Physical activity (10 items)
- Physical limitations (4 items)
- Physical pain (2 items)
- Perceived health (5 items)

Mental component:

- Life and relationships with others (2 items)
- Mental health (5 items)
- Limitations due to mental state (3 items)
- Vitality (4 items)

Other:

- Change in perceived health (1 question): The respondent is asked to rate his or her current health status by comparing it to his or her health status 1 year earlier

#### Study Stopping Rules

Research may be suspended or stopped by the sponsor in consultation with the coordinator or the Ethics Committee (Comité de Protection des Personnes) for insufficient patient recruitment.

#### Premature Withdrawal From the Study

Premature withdrawal exits may be justified by the following reasons: withdrawal of consent, lost from sight, patient no longer wishing to wear the hearing aid (considered a failure), death, and loss of hearing aid.

Patients participating in the research can withdraw their consent and ask to leave the trial at any time and for any reason, without having to justify themselves, without losing their right to be treated by their doctor.

Patients will continue to participate in the study whether the dose of platinum-based chemotherapy is modified following an adverse event and according to the recommendations of the patient’s primary care physician, and whether the administration of platinum-based chemotherapy is permanently discontinued during the study.

### Projected Study Schedule

The estimated duration of recruitment is 18 months. The estimated duration of each participant from the signing of the consent until 6 months after equipment is 8 months maximum. The estimated duration of the project is 36 months, including the recruitment period, the follow-up period, quality control (monitoring), and statistical analysis.

### Data Collection

Data on hearing aid usage, adverse events, and satisfaction will be collected through standardized electronic case report forms and stored securely on the CleanWeb platform. Feasibility will be assessed by the proportion of patients who accept and adhere to the hearing aid intervention, while acceptability will be evaluated using patient-reported satisfaction scores and qualitative feedback collected during the final visit (V3).

**Figure 1.**
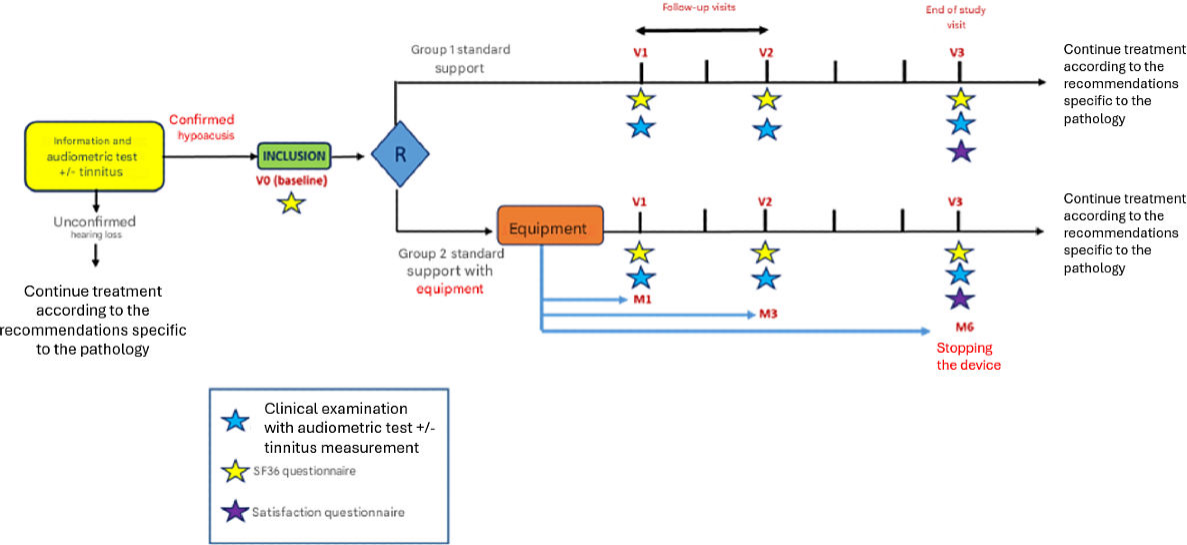
Timeline of the study.

Figure 1 describes the timeline of the study. Table 1 gives a summary of the investigations performed during the study.

**Table 1.** Summary table of investigations.

Visits	Assessment of inclusion (visit 0)	Follow-up report (visit 1 at the 1-month follow-up)	Follow-up report (visit 2 at the 3-month follow-up)	End-of-study assessment (visit 3 at the 6-month follow-up)
Research data				
For all patients				
Inclusion/noninclusion criteria	X			
Signed informed consent	X			
Randomization	X			
SF-36^a^ quality of life questionnaire	X	X	X	X
Satisfaction questionnaire				X
For group 2 patients only				
Hearing aid reference	X			
Worn time of the hearing aid		X	X	X
Adverse effects of wearing a hearing aid		X	X	X
Collection of hearing aid malfunctions		X	X	X
Data collected as part of care (for all patients)				
ENT^b^ consultation	X	X	X	X
Audiogram	X	X	X	X
Tinnitus	X	X	X	X
Adverse events		X	X	X
Administration of platinum salt treatment		X	X	X

a SF-36: 36-Item Short-Form Health Survey.

b ENT: ear nose and throat.

### Primary Outcomes

The quality of life of patients with ototoxicity due to platinum-based chemotherapy treatment will be assessed using the SF-36 score. The questionnaire consists of 36 questions divided into 8 domains: functional capacity, physical aspects, pain, general health, vitality, social aspects, emotional aspects, and mental health. The calculation of the score is a scale from 0 to 100, where 0 corresponds to a worse quality of life and 100 to a better quality of life [39,41].

In this study, quality of life will be assessed by an overall score calculated by the average of the observed scores across the 8 domains. It will be assessed at baseline before randomization in order to measure the initial score of the patient’s quality of life, then during the follow-up after randomization: M1, M3, and M6 of follow-up.

### Secondary Outcomes

Hearing and tinnitus occurrence in patients with chemotherapy-induced ototoxicity will be assessed by Hertz/Decibel encryption using the Interacoustics Diagnostic Audiometer AD229b.

The hearing of patients will be characterized by 2 modalities: aggravation or stagnation. The appearance of tinnitus will be measured by an incidence rate.

Satisfaction will be measured at 6 months (end of the study) by a numerical scale from 0 to 10 (0: not satisfied; 10: very satisfied).

Patients’ adherence to hearing aid use in cases of ototoxicity due to chemotherapy treatment will be assessed by an adherence rate which is defined by the proportion of patients who have completed the 6 months of protocol follow-up with hearing aid use among patients randomized to the experimental group (hearing aid).

## Results

Experiments are underway with patients already enrolled, but the study is actively recruiting other participants. The protocol was launched on November 23, 2023, and data collection is ongoing and will be completed once all patients have completed the protocol. At least 27 patients have been recruited to date. The date of publication of the results is not yet known.

## Discussion

The aim of this study is to determine if hearing aids improve quality of life in platinum-related hearing loss.

Numerous papers in the literature indicate that hearing loss can be followed by severe psychosocial consequences with a decrease in mood and quality of life [42]. In a general population, a study concludes that half of the 158 adults with hearing loss recruited before hearing aid fitting want to renew their hearing aid [43]. In addition, the authors note that the quality of life of patients with hearing loss was like that of the general population, but poorer than in many serious chronic diseases.

Improvement of hearing loss and tinnitus can significantly improve patients’ daily lives and better tolerability and adherence to treatment [44,45]. In the long term, we can expect a proposal for systematic hearing aids for patients suffering from ototoxicity of chemotherapy. The aim of the study is to offer patients under chemotherapy a better quality of life. A study reveals that only 33% of health professionals know how to manage the ototoxic effects of chemotherapy [2]. The protocolization of care by hearing aid would allow a democratization of the practice in health institutes.

The heterogeneity of ototoxicity induced by platinum-based chemotherapy, along with potential clinical and environmental covariates, can significantly affect the outcomes and interpretation of a clinical study evaluating the effectiveness of hearing aids in affected patients. Variability in the severity and pattern of hearing loss—driven by factors such as cumulative dose, type of platinum agent, age, renal function, genetic susceptibility, and co-exposure to other ototoxic agents—may lead to wide interindividual differences in auditory deficits. This can increase data dispersion, reduce statistical power, and obscure the true impact of hearing aids. Additionally, confounding variables such as cognitive status, socioeconomic factors, comorbidities, prior hearing aid use, and time since treatment may influence both hearing loss severity and responsiveness to auditory rehabilitation, thereby threatening the study’s internal validity. These factors also pose challenges to external validity, as generalizing findings for a broader patient population becomes more complex.

Ototoxicity induced by platinum-based chemotherapy is generally irreversible or only partially reversible. Hearing loss typically develops during or shortly after treatment and then stabilizes. Consequently, assessment intervals at 1 month (M1), 3 months (M3), and 6 months (M6) posttreatment are strategically chosen to capture the onset, progression, and plateau phases of ototoxicity. These points enable time evaluation of hearing impairment and the effectiveness of rehabilitative interventions such as hearing aids.

Extending follow-up to 12 months (M12) is considered less informative, as significant further recovery or deterioration is uncommon beyond 6 months. Additionally, longer follow-up periods may increase patient attrition and study burden without providing meaningful additional data. Therefore, focusing on the first 6 months posttreatment balances scientific rigor with practical considerations, ensuring robust and clinically relevant data collection.

## Abbreviations

ANCOVA: analysis of covariance
ENT: ear, nose, and throat
SF-36: 36-Item Short-Form Health Survey
